# Visible-light-induced superhydrophilicity of crystallized WO_3_ thin films fabricated by using a newly isolated W^6+^ complex salt of citric acid†

**DOI:** 10.1039/d2na00717g

**Published:** 2023-03-07

**Authors:** Taichi Murayama, Mitsunobu Sato, Hiroki Nagai, Eiko Yasui

**Affiliations:** a Electrical Engineering and Electronics Program, Graduate School of Engineering, Kogakuin University of Technology and Engineering Hachioji Tokyo 192-0015 Japan nagai@cc.kogakuin.ac.jp; b Department of Applied Physics, School of Advanced Engineering, Kogakuin University of Technology and Engineering Hachioji Tokyo 192-0015 Japan; c Department of Chemistry and Life Science, School of Advanced Engineering, Kogakuin University of Technology and Engineering Hachioji Tokyo 192-0015 Japan

## Abstract

Transparent tungsten trioxide thin films, which demonstrated visible-light (Vis-light)-induced superhydrophilicity, with thicknesses of 100–120 nm, adhesion strengths greater than 49 MPa, bandgap energies of 2.8–2.9 eV, and haze values of 0.4–0.5%, were fabricated using a solution-based process on quartz glass substrates. The precursor solution was prepared by dissolving a W^6+^ complex salt isolated from a reacted solution of tungstic acid, citric acid, and dibutylamine in H_2_O, in ethanol. By heating the spin-coated films in air for 30 min at temperatures higher than 500 °C, crystallized WO_3_ thin films were obtained. The O/W atomic ratio was evaluated to be 2.90, based on the peak area analysis of X-ray photoelectron spectroscopy spectra of the thin-film surfaces, indicating the co-presence of W^5+^ ions. The water contact angle on film surfaces, which was approximately 25° prior to light irradiation, decreased to less than 10° upon irradiation with 0.06 mW cm^−2^ Vis-light for only 20 min at 20–25 °C and a relative humidity (RH) of 40–50%. By comparing the contact angle changes at RH values of 20–25%, it was revealed that the interaction between ambient water molecules and the partially O-deficient WO_3_ thin films plays an important role in achieving photoinduced superhydrophilicity.

## Abstract 2

Transparent tungsten trioxide thin films, which demonstrated visible-light (Vis-light)-induced superhydrophilicity, were fabricated using a solution-based process on quartz glass substrates.

## Graphical abstract

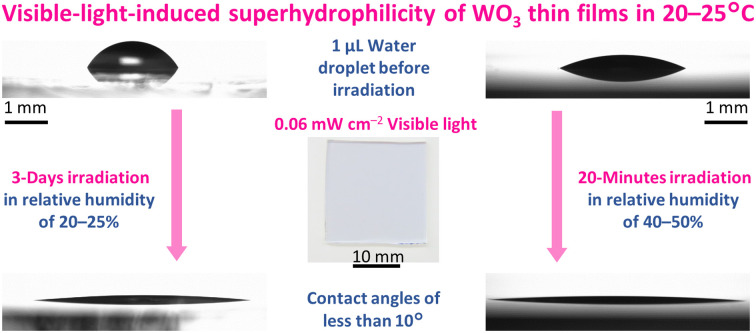

## 1. Introduction

Crystallized tungsten trioxide (WO_3_) thin films with photoinduced hydrophilicity as useful nanoscale materials on ceramic substrates using dry or wet processes have been fabricated by several research groups.^1–4^ For obtaining high-quality thin films that are small in size, dry processes such as sputtering and chemical vapor deposition are generally suitable; however, they require complicated and expensive equipment. In contrast, wet processes that can be applied to large-area substrates are relatively simple and inexpensive. Therefore, WO_3_ thin film formation has been investigated using various wet processes such as sol–gel,^1,5,6^ spray,^7^ electroplating,^8^ and solvothermal methods.^9^ Those methods use various starting materials including sodium tungstate (Na_2_WO_4_),^1,10,11^ tungsten hexachloride (WCl_6_),^5,7,12,13^ and tungstic acid (H_2_WO_4_).^14,15^ When Na_2_WO_4_ was used, the authors replaced Na^+^ ions with H^+^, because Na^+^ ions tend to remain as an inevitable impurity in the product after necessary heat treatment. Also, WCl_6_ is unstable in moist air and requires special care in handling. Additionally, H_2_WO_4_ is insoluble in water, so hydrofluoric acid or concentrated hydrochloric acid must be added to prepare a homogeneous solution. Thus, it is generally difficult to find raw materials with easy handling.

The superhydrophilicity of WO_3_ thin films induced by ultraviolet-light (UV-light) irradiation was widely reported^2,15,16^. On the other hand, there is still only one report on visible-light (Vis-light)-induced superhydrophilicity of WO_3_ films. An approximately 1 μm-thick film was fabricated on silica glass substrates using a dry process, DC magnetron sputtering, and subsequent heat treatment.^17^ The water contact angle on the thick film depended on the surface roughness, and the most hydrophilic film had a surface roughness of 41.4 nm with no report on the haze value. Additionally, there are no reports on the photoinduced superhydrophilicity of transparent WO_3_ thin films *via* wet processes, to the best of our knowledge. An inexpensive method to fabricate transparent and low-haze WO_3_ thin films adhered strongly onto glass substrates, which have Vis-light-induced superhydrophilicity, is important for practical use under daily light.

The molecular precursor method (MPM) used in this study is one of the wet processes and useful for fabricating metal oxide, metal, and metal phosphate thin films, usually in the thickness range of several tens to several hundred nanometers. A typical MPM uses a precursor solution consisting of an alkylammonium salt of a stable metal complex in which the central metal ion is bound to a multidentate ligand such as ethylenediamine-*N*,*N*,*N*′,*N*′-tetraacetic or nitrilotriacetic acids. In many cases, the precursor solution consists of relatively stable complexes dissolved at the molecular level into ethanol and can be used as a stable coating solution under ambient conditions.^18,19^ The design of appropriate combinations of countercations and metal complexes having discrete molecular structures is essential for the nanoscale thin films of densified metal compounds.

In this study, the dibutylammonium salt of a W^6+^ complex with a citrate ligand was prepared from easy-to-handle raw materials and fully characterized. The stable precursor solution, which was obtained by dissolving the salt into ethanol, could be stored under ambient conditions for at least 3 months and spin-coated onto a quartz glass substrate. The crystallized transparent WO_3_ thin films with low haze values were successfully fabricated by subsequent heating of the precursor film at temperatures higher than 500 °C. The unprecedented Vis-light-induced superhydrophilicity that occurred under very short-term and weak light irradiation onto the crystallized WO_3_ thin films is reported, along with the important role of the co-present W^5+^ ions and oxygen deficiency which were elucidated by XPS analyses.

## 2. Experimental

**Fig. 1:**
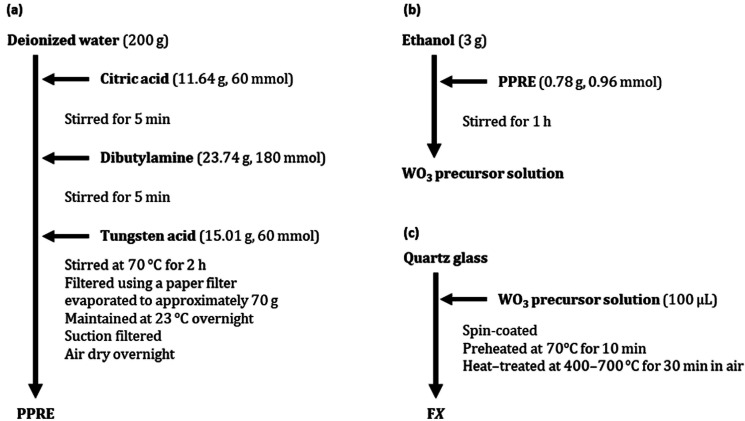
The experimental routes for (a) the isolated PPRE and (b) the WO_3_ precursor solution dissolving the powder and (c) the procedures for coating and heat treatment.

The preparation routes from raw materials to the isolated powder of the precursor complex (PPRE) and the WO_3_ precursor solution dissolving the powder, and the procedures for coating and heat treatment are shown in Fig. 1. The obtained thin films heat treated at 400, 500, 600 and 700 °C for 30 min in air are denoted as F*X*, where *X* represents each heating temperature. The details and other materials used in this study are given in ESI S1.†

### 2.1. Chemical characterization of dibutylammonium salt of the W^6+^ complex with citrate ligands

A portion (100 mg) of PPRE was dried in a vacuum for 1 h before its chemical characterization. The IR spectrum of PPRE was acquired from a disk diluted with KBr using an FT/IR 4700 spectrophotometer (JASCO Corp., Tokyo, Japan). 200 mg of KBr as a diluent was ground in a mortar and compressed to form a pellet for reference. Separately, 3 mg of PPRE was mixed with 200 mg of pre-ground KBr. The mixed powders were thoroughly ground in a mortar and compressed to form a sample pellet. The spectrum of the pellet was measured in the range of 400–4000 cm^−1^ using 16 data acquisition mode. The thermogravimetric analysis (TG) and differential thermal analysis (DTA) curves of 8.0 mg of PPRE were measured using a TG-2000s instrument (BRUKER, Massachusetts, USA). α-Al_2_O_3_ powder (10 mg) in a Pt crucible was used as a reference. The temperature was increased from 25 to 1000 °C at a rate of 2 °C min^−1^ and an air flow rate of 0.1 L min^−1^. Elemental analysis of PPRE was performed using a 2400 Series II CHN analyzer (PerkinElmer, Massachusetts, USA). ^1^H- and ^13^C-nuclear magnetic resonance (NMR) spectra of PPRE in C_2_D_5_OD were measured using a JNM-ECZ400S spectrometer at 400 MHz (JEOL, Tokyo, Japan). PPRE (10.5 mg) was dissolved in 1 mL of C_2_D_5_OD, and tetramethylsilane was used as an internal standard.

### 2.2. Structural and chemical characterization of thin films

The X-ray diffraction (XRD) patterns of the four thin films, F400, F500, F600, and F700, were measured in a step scan mode using an X-ray diffractometer, SMARTLAB (Rigaku, Tokyo, Japan), with Cu*-*Kα rays generated at 45 kV and 200 mA. Parallel beam optics with an incident angle of 0.3° in the 2*θ* range of 20–60°, a step width of 0.01°, and a scan speed of 0.2° min^−1^ were used.

The X-ray photoelectron spectroscopy (XPS) spectra of the three thin films, F500, F600, and F700, were measured using a JPS-9030 spectrometer (JEOL Ltd., Tokyo, Japan) using non-monochromatic Mg-Kα X-ray radiation (1486.6 eV) and a step width of 0.1 eV. The binding energies of the XPS spectra were corrected with respect to the C 1s peak at 284.6 eV. Peak fitting of XPS spectra was performed using Origin peak-fitting module software (Microcal Origin, version 9.0, Massachusetts, USA).

### 2.3. Surface morphology, film thickness, and adhesion strength of thin films

The surface morphologies of the four thin films, F400, F500, F600, and F700, were observed *via* field-emission scanning electron microscopy (FE-SEM) using a JSM-6701F microscope (JEOL Ltd., Tokyo, Japan) at an accelerating voltage of 20 kV. The average grain size of each thin film was determined using five randomly selected grains. The thickness of the four thin films was measured using a stylus profilometer (DEKTAK-DXT-E, BRUKER, Massachusetts, USA). For the sample preparation, a portion of the substrate was masked to expose the substrate. The level differences at five positions between the substrate and heat-treated thin film were determined for each sample. The average film thickness was computed excluding the highest and lowest values. The adhesion strengths of F500, F600, and F700 on the quartz glass substrate were examined using a stud-pull adhesion test. A stand pin, P/N901106, with an internal diameter of 2.7 mm was attached to the film using epoxy glue and placed in an oven at 150 °C for 1 h. The test was subsequently performed by pulling the stand pin with a load ranging from 0 to 100 kg at a rate of 2.0 kg s^−1^.

### 2.4. Optical characterization of thin films

The transmittance spectra of the four thin films, F400, F500, F600, and F700, were measured in the range of 200–1100 nm using a double-beam spectrophotometer UV-1900i (SHIMADZU, Kyoto, Japan). Clean quartz glass was used as a reference. The Tauc plot obtained using the transmittance data was used to estimate the optical bandgap of the three thin films, F500, F600, and F700, using the relationship between *x* and *y* as expressed using the following eqn (1)–(3), assuming an indirect bandgap (*n* = 2).1
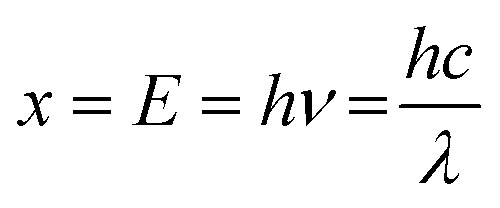

2 *y* = (*hva*)^1/*n*^

3
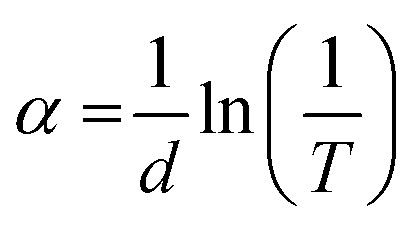
In these equations, *E* is photon energy, *h* is the Planck constant, *c* is 3.0 × 10^8^ m s^−1^, *λ* is wavelength, *T* is transmittance, *d* is film thickness, and *α* is the absorption coefficient at *λ*.

The haze values of the three thin films, F500, F600, and F700, were measured using a haze meter COH7700 (Nippon Denshoku Industries Co., Ltd., Tokyo, Japan). The average haze value was calculated by excluding the highest and lowest values.

### 2.5. Photoinduced hydrophilicity of thin films

The contact angles of a 1.0 μL water droplet on each surface of the four thin films, F400, F500, F600, and F700, were measured using a contact angle meter (FACE, Kyowa Interface Science, Saitama, Japan). The Vis-light of 0.06 mW cm^−2^ and UV-light of 0.01 mW cm^−2^ were irradiated from a light-emitting-device, ECL-111 (KOIZUMI, Osaka, Japan). The Vis-light and UV-light intensities were monitored using solar power meters, DT-1307 (MK Scientific, Kanagawa, Japan) and CL-H1-365-9-1 (Asahi Spectra, Tokyo, Japan), respectively.

First, the thin film samples were kept in the dark in the temperature range of 20–25 °C and relative humidity (RH) range of 20–25% for three days before measurement. The water contact angle measurements were performed once daily. Light irradiation was performed for only the first three days. After this, the samples were stored in the dark for one day, and thereafter light irradiated for one day. These procedures were repeated for up to nine days. The contact angles of the water droplets were measured in the temperature range of 20–25 °C and RH range of 20–25%.

Second, the thin film samples were kept in the dark in the temperature range of 20–25 °C and RH range of 40–50% for three days before measurement. The water contact angle was measured before and after light irradiation for 10, 20, 30, and 60 min because the water contact angle before irradiation was sufficiently small. After light irradiation for 60 min, the samples were stored in the dark for one day. Thereafter, the contact angles of the water droplets were measured at 20–25 °C and in a RH range of 40–50%.

## 3. Results

### 3.1. Chemical characterization of dibutylammonium salt of the W^6+^ complex with a citrate ligand

The results of the elemental analysis of PPRE obtained by the reaction of tungstic and citric acids in the presence of dibutylamine in water were: C, 43.5%; H, 8.1%; N, 5.1%. (calc. for WC_30_H_67_N_3_O_11_: C, 43.4%; H, 8.1%; N, 5.1%). The computed values for WC_30_H_67_N_3_O_11_ are given in parentheses and are consistent with the experimental data. The yield of the compound was determined based on the mass of the product (23.0 g, 46%).

In the FT-IR spectrum of PPRE (Fig. S1†), two intense bands observed at 1583 and 1635 cm^−1^ can be assigned to the asymmetric stretching mode, *ν*_as_(COO^−^), of the deprotonated carboxyl groups in the citrate ligand.^20^ The other two clear bands observed at 1384 and 1408 cm^−1^ can be assigned to the symmetric stretching mode, *ν*_s_(COO^−^), of the deprotonated carboxyl groups in the citrate ligand.^20^ These peak positions assigned to the dissociated carboxyl groups were shifted from those of free H_4_cit reported at 1706, 1745, and 1753 cm^−1^.^21,22^

In the ^1^H-NMR spectrum of PPRE, a doublet doublet peak was observed at 2.62 ppm (Table S1†). This peak can be assigned to the hydrogen atoms of the methylene group in the citrate ion. In addition, two triplet peaks were observed at 0.96 and 2.96 ppm. Each peak in the order from the higher magnetic field can be assigned to the hydrogen atom of the methyl group and that of the methylene group bonded to the nitrogen atom of the dibutylammonium ion. Furthermore, sextet and quintet peaks were observed at 1.41 and 1.71 ppm. Each peak in the order from the higher magnetic field can be assigned to the hydrogen atom of the methylene group linked to the methyl group and that of the adjacent methylene group of the dibutylammonium ion, respectively. In the ^13^C-NMR spectra obtained using the proton homodecoupling mode, two singlet peaks were observed at 45.51 and 85.91 ppm (Table S2†). These peaks can be assigned to the carbon atoms of the methylene group and those linked to the hydroxy group of citric acid, respectively.^20^ In addition, two singlet peaks were observed at 177.37 and 186.29 ppm. These peaks can be assigned to the carbon atoms of two equivalent carboxy groups and the carbon atom of one carboxy group connecting to the tertiary carbon atom, respectively.^20^ Four additional singlet peaks were observed at 13.99, 20.73, 28.84, and 48.02 ppm. Each peak in the order from the higher magnetic field can be assigned to the carbon atom of the methyl group, that of the methylene group linked to the methyl group, that of the adjacent methylene group, and that of the methylene group bonded to the nitrogen atom of the dibutylammonium ion, respectively. These results demonstrated that the chemical formula of the product was (Bu_2_NH_2_)_3_[WO_3_(Hcit)]·H_2_O.

**Fig. 2:**
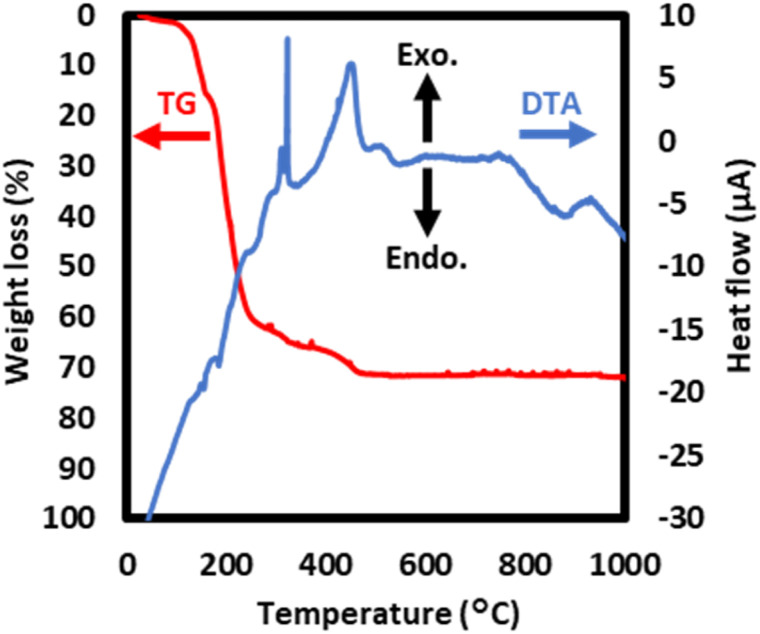
TG-DTA curves of PPRE. The measurement temperature was increased from 25 to 1000 °C at a rate of 2 °C min^−1^ and an air flow rate of 0.1 L min^−1^.

The TG and DTA curves of PPRE exhibited mass losses and endothermic and exothermic peaks at several heating temperatures, respectively, as shown in Fig. 2. A mass loss of 65% accompanied by both endothermic and exothermic peaks in the temperature range of 100–330 °C can be attributed to the removal of hydrated water and dibutylamine molecules. The TG curve shows that PPRE decomposed completely up to 450 °C, with a final residue of 28 mass%.

### 3.2. Crystal structure and chemical characterization of thin films

**Fig. 3:**
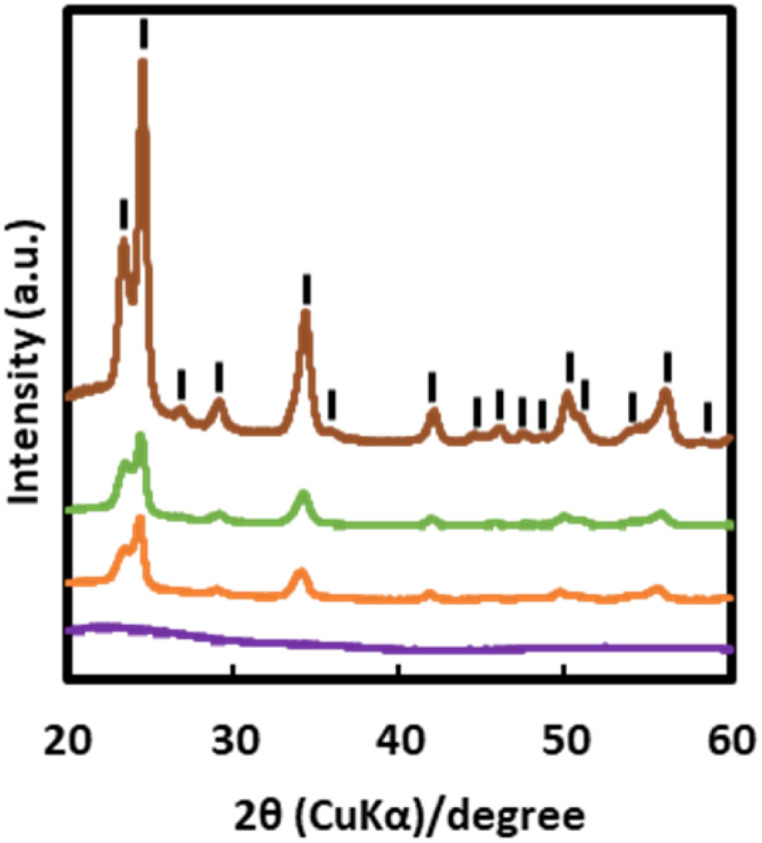
XRD patterns of the thin films obtained by heating at several temperatures (purple: F400, orange: F500, green: F600, and brown: F700) fabricated on a quartz glass substrate in air. Parallel beam optics with an incident angle of 0.3° in the 2*θ* range of 20–60° were used. The peaks are denoted as follows: |WO_3_.

The XRD patterns of F400, F500, F600, and F700 are shown in Fig. 3. The XRD pattern of F400 exhibited no clear peak. The XRD patterns of F500, F600 and F700 can be assigned to WO_3_ crystals that are either triclinic (ICDD no. 01-071-0305),^23^ monoclinic (ICDD no. 01-083-0950)^24^ or orthorhombic (ICDD no. 01-089-4477).^25^ The XRD peak positions of F500, F600, F700 and ICDD data are shown in Table S3 and S4.†

**Fig. 4:**
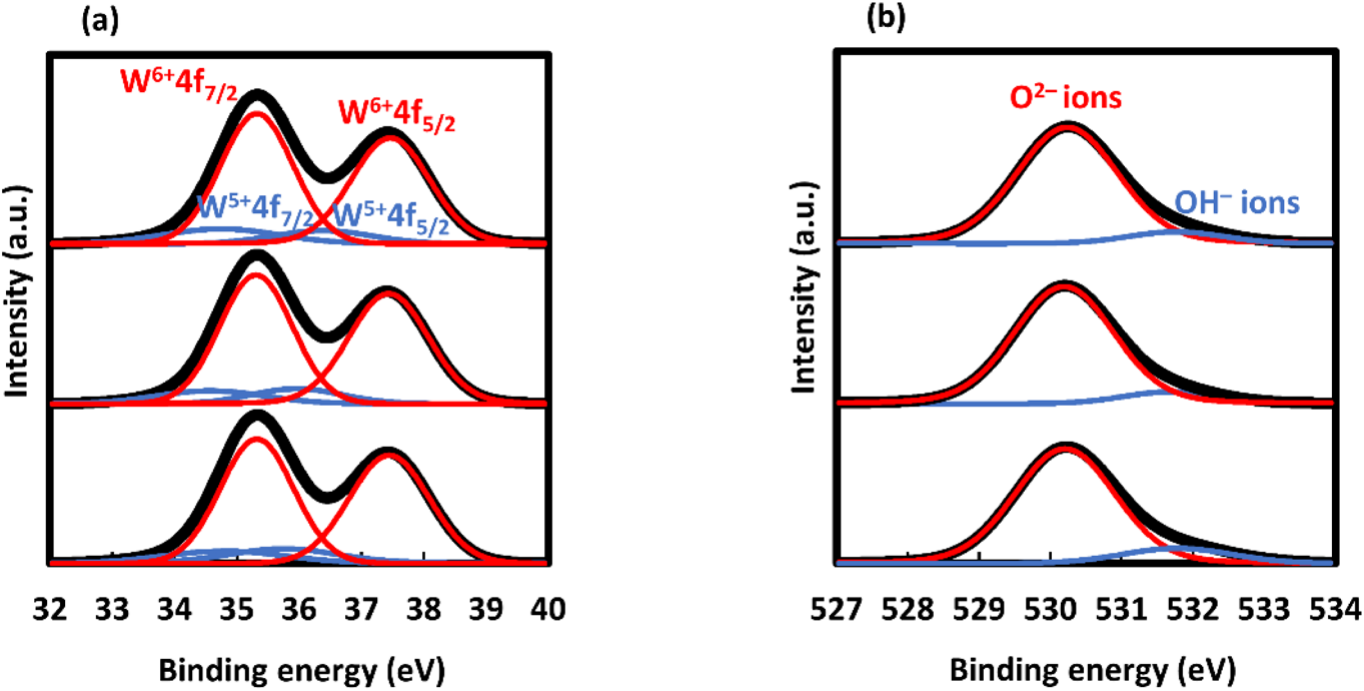
High-resolution XPS spectra of (a) W 4f and (b) O 1s of the thin films obtained by heating at several temperatures (F500 (bottom lines), F600 (middle lines), and F700 (top lines)). The thick bold line indicates the original XPS data, whereas the colored line indicates the theoretically fitted curve assuming a Voigt distribution.

The XPS spectra of F500, F600, and F700 and those after Vis-light irradiation in a RH range of 40–50% for 20 min are shown in Fig. 4 and S2,† along with the deconvoluted peaks. The peaks in the energy ranges of 32–40 eV and 527–534 eV were deconvoluted into four and two components, respectively (Tables S5 and S6†). The peaks assigned to W 4f_7/2_ and W 4f_5/2_ for W^6+^ ions were observed at 35.3 and 37.4 eV, respectively, and those for W^5+^ ions were observed at lower energies at 34.7 and 36.1 eV, respectively.^26^ The peaks attributed to the O^2−^ and OH^−^ ions were observed at 530.2 and 531.7 eV, respectively.^27,28^ The O/W atomic ratios determined from the peak area ratio, (the area of the O 1s peak attributed to O^2−^ ions/O 1s of the relative factor)/(the area of the W f_7/2_ peak of W^6+^ ions/W f_7/2_ of the relative factor), were 2.90 for F500, F600, and F700 before and after Vis-light irradiation in a RH range of 40–50% for 20 min, according to a computation method used in a reference.^26^ The relative factors used for O 1s and W f_7/2_ were 10.9553 and 24.2057, respectively, and obtained from the XPS operating software (SpecSurf Acquisiton, JEOL Ltd., Tokyo, Japan). Additionally, the peak area ratios of OH^−^ ions in oxygen species after Vis-light irradiation in a RH range of 40–50% for 20 min before irradiation were 0.38, 0.45 and 0.73 for F500, F600, and F700 respectively.

### 3.3. Surface morphology, film thickness, and adhesion strength of thin films

**Fig. 5:**
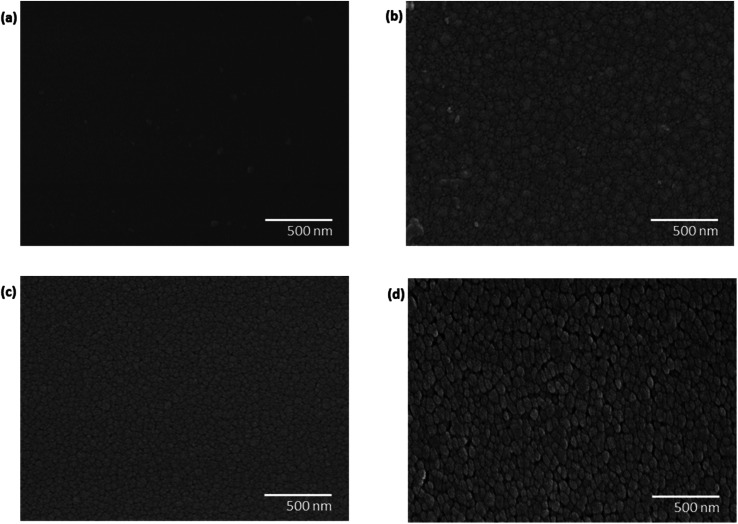
FE-SEM images of the thin films obtained by heating at several temperatures ((a) F400, (b) F500, (c) F600, and (d) F700) adhered on a quartz glass substrate.

FE-SEM images of F400, F500, F600, and F700 are shown in Fig. 5. The FE-SEM images show that the surfaces of all the thin films are smooth. The average grain sizes of F500, F600, and F700 were 100, 63, and 124 nm, respectively. The film thicknesses of F400, F500, F600, and F700 were 150, 100, 120, and 120 nm, respectively, with an experimental error of 10 nm. The profiles in the measurement of film thicknesses for F400, F500, F600, and F700 are shown in Fig. S3.† The adhesion strength of F500 on the quartz glass substrate was 49 ± 6 MPa, whereas that of F600 and F700 was greater than 70 MPa. The hysteresis curves in the measurement of adhesion strength for F400, F500, F600, and F700 are shown in Fig. S4.†

### 3.4. Optical properties of thin films

**Fig. 6:**
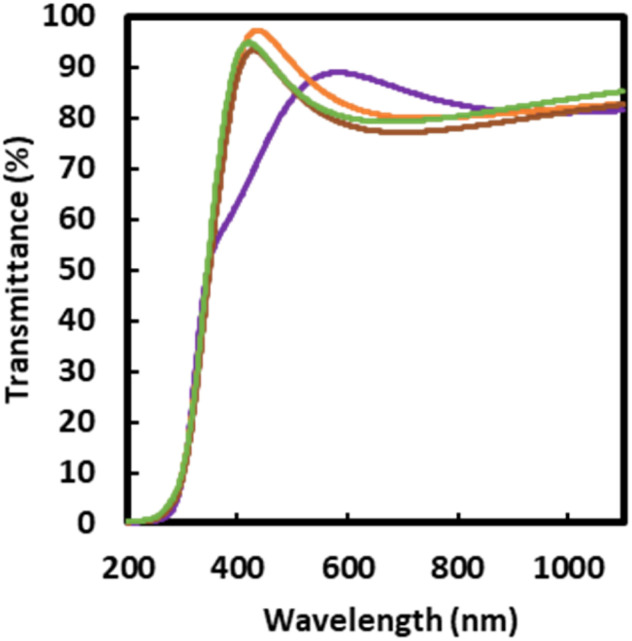
Transmittance spectra of the thin films obtained by heating at several temperatures (F400 (purple), F500 (orange), F600 (green), and F700 (brown)). The quartz glass substrate was used as a reference for the thin film transmittance measurements.

The transmittance spectra of F400, F500, F600, and F700 are shown in Fig. 6. The transmittance of all thin films was higher than 70% in the visible region. The optical bandgaps obtained from the Tauc plots of F500, F600, and F700 were 2.8, 2.9, and 2.8 eV, respectively.

The average haze values of F500, F600, and F700 were 0.5%, 0.4%, and 0.4%, respectively, with an experimental error of 0.1%.

### 3.5. Photoinduced hydrophilicity of thin films

**Fig. 7:**
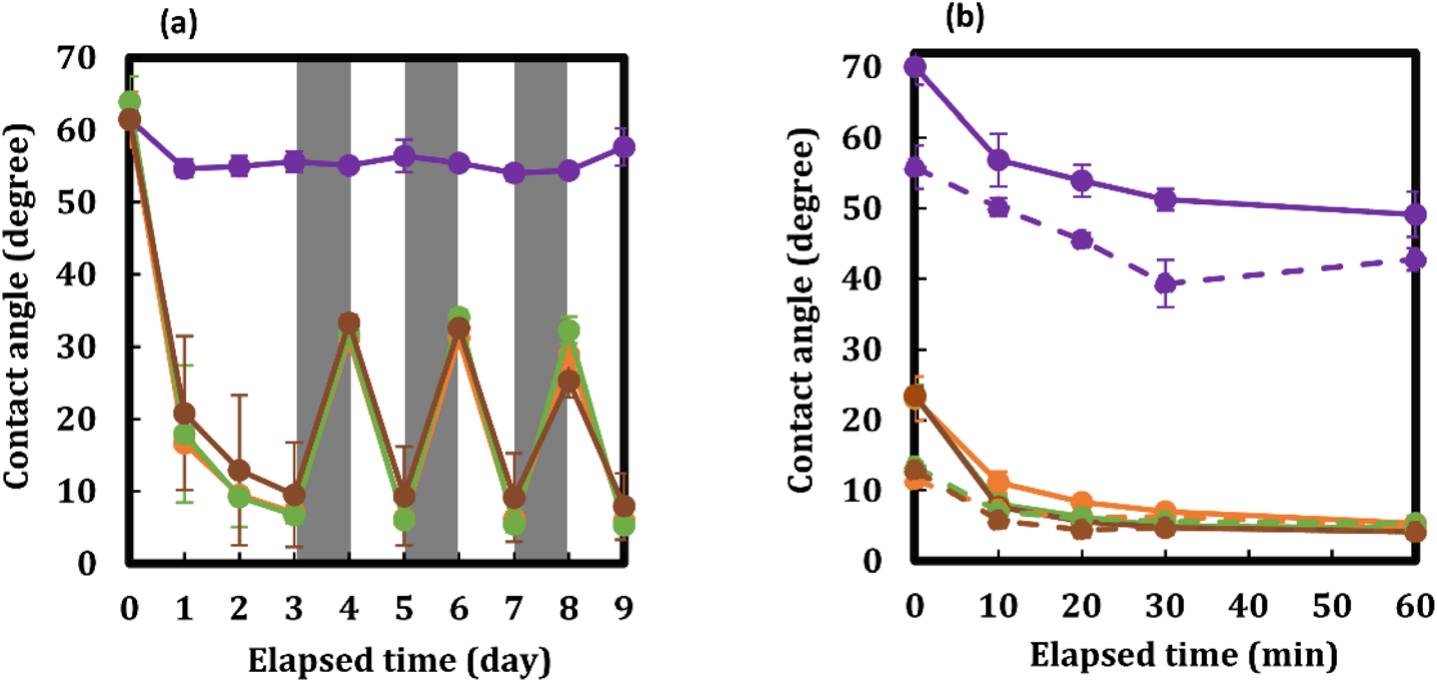
Water contact angles of the thin films obtained by heating at several temperatures, (F400 (purple), F500 (orange), F600 (green), and F700 (brown)) under visible light in the temperature range of 20–25 °C and RH ranges of (a) 20–25% and (b) 40–50%. (a) The visible light irradiation (white areas) and storage in the dark (grey areas) were repeated. (b) The solid and dashed lines represent visible light irradiation of the films after heat treatment and after one day of storage in the dark, respectively.

In Fig. 7(a), the changes in the water contact angles on F400, F500, F600, and F700, which were maintained in a RH range of 20–25% for three days before the measurement, are presented along with the error bar. The changes in the contact angle on F400 were less than 10° even after continuous light irradiation for three days. In contrast, the contact angles of F500, F600, and F700 decreased rapidly and the values of approximately 60° before irradiation reached 7° ± 1°, 7° ± 1°, and 10° ± 7°, respectively, after irradiation for the first three days (Table S7†). The values of these three thin films changed repeatedly from 30° to 10° by keeping them under dark conditions for one day and under Vis-light irradiation for one day, at an identical low RH for more than one week.

In Fig. 7(b), the changes in the water contact angles on F400, F500, F600, and F700, which were maintained in a RH range of 40–50% for three days before measurement, are presented along with the error bar. The degree of decrease, even after light irradiation for only 60 min, was more than twice that of those irradiated for more than one day (Fig. 7). Light irradiation for only 20 min in this RH range on F500, F600, and F700, whose water contact angles were approximately 23° after being kept in the dark for three days, reduced each contact angle rapidly to 8–6° with an experimental error of 1° (Table S8†).

Fig. 7(b) shows the changes in the water contact angles on F500` F600, and F700, which were measured again after they reached the superhydrophilic state once by Vis-light irradiation for 60 min and subsequently left in the dark in a RH range of 40–50% for one day. It was demonstrated that the values on these thin films decreased to 7–6° for only 10 min of Vis-light irradiation after the values increased to approximately 13° when left in the dark.

## 4. Discussion

### 4.1. Identification of the precursor complex salt

Li *et al.* reported the synthesis of (NH_4_)_4_[WO_3_(cit)]·2H_2_O by the reaction of (NH_4_)_2_WS_4_ with two molar amounts of (NH_4_)_3_(Hcit) in water, where cit and Hcit represent tetravalent and trivalent anionic ions derived from citric acid, respectively.^20^ They observed that the four proton ions dissociated from three carboxy groups and one hydroxy group in citric acid (H_4_cit), yielding a tetravalent anionic ligand. In contrast, (Bu_2_NH_2_)_3_[WO_3_(Hcit)]·H_2_O was isolated in this study by the reaction of equimolar amounts of WO_3_ and citric acid with three times the molar amount of dibutylamine in water. In the IR spectrum, the peak shifts of the carboxyl groups in citric acid were clearly observed because of deprotonation of the groups. Additionally, the peak positions of both vibrations, *ν*_as_(COO^−^) and *ν*_s_(COO^−^), were observed to be more than 15 cm^−1^ higher than those for (NH_4_)_4_[WO_3_(cit)]·2H_2_O reported by Li *et al.*;^20^ 1565 and 1614 cm^−1^ for *ν*_as_(COO^−^), and 1368 and 1390 cm^−1^ for *ν*_s_(COO^−^). These peak shifts may be due to the strain relaxation of the carboxyl groups bonded to the central W^6+^ ion because the protonated hydroxy group is free from coordination in the current complex. Additionally, the TG-DTA results were consistent with the elemental analysis of the powder, assuming that citric acid acts as a trivalent anionic ligand, Hcit. From the NMR spectra in deuterated ethanol solution in which the powder was dissolved, it was also elucidated that the three carboxyl groups were coordinated to the central W^6+^ ion in the precursor solution.

### 4.2. Phase transition and oxygen-deficient formation in thin films

The TG-DTA curves of the isolated complex salt, (Bu_2_NH_2_)_3_[WO_3_(Hcit)]·H_2_O, indicated an endothermic peak, which could be due to the phase transition at approximately 550 °C with no mass decrease (Fig. 2). Because the NMR spectra of the dissolved complex salt demonstrate that the component in the ethanol solution is identical to that of the isolated powder, the thermal reaction of the precursor film obtained through spin-coating of the precursor solution is identical to that of the isolated complex. Therefore, it is clear that the thermal reaction of the precursor film on the quartz glass substrate involves phase transition in the corresponding temperature region. The crystal systems of WO_3_ thin films that were formed on glass substrates at 500 °C through both dry and wet processes are monoclinic,^3,7^ triclinic,^14,16,17^ hexagonal,^29^ and orthorhombic.^5,26^ Ramana *et al.* reported that the crystal structure and oxygen-deficiency level of hexagonal WO_3_ thin films depend on the fabrication and annealing conditions.^29^ Due to the similarity of peak positions in several crystal systems, it is very difficult to unambiguously assign all peaks to specific crystal systems from only the XRD patterns of current WO_3_ thin films. On the other hand, the degree of hydrophilization before and after the phase transition is not greatly affected, so we will investigate the phase transition revealed by thermal analysis in detail separately in the future.

Because the precursor film is composed of a metal complex salt containing organic compounds, a certain amount of oxygen in the furnace should be used to burn the organic compounds during the removal of these organic compounds to form WO_3_, resulting in an oxygen deficiency. Owing to this heat reaction mechanism, oxygen deficiency consequently occurred in the WO_3_ thin films, even though the atmosphere was air and not Ar as reported in a previous study.^26^ Therefore, the chemical formula of the fabricated tungsten oxide thin film surfaces was determined to be WO_2.90_ from the XPS results independent of the heating temperature (Fig. 4).

### 4.3. Effects of crystal growth and phase transition in thin films

The film thicknesses of F600 and F700 were 120 nm, which were 20 nm thicker than F500. Generally, crystal growth at higher temperatures enhances densification, and film thickness tends to decrease. Therefore, the 20% increase in thickness from F500 to F600 and F700 suggests that the phase transition prevents densification and has the opposite effect. This opposite effect by the phase transition seems to be due to the grain decay that yielded several interstices in the grain boundaries, as the grain size of F600 is 40 nm less than that of F500 (Fig. 5). It was also observed that the interstices formed during the phase transition were maintained, although the grains of F700 were grown by heat treatment at the high temperature. In contrast, the fact that the adhesion strength of the thin films on the quartz glass substrate is independent of the effects of crystal growth and phase transition indicates the usefulness of WO_3_ thin film formation using the proposed method.

### 4.4. Vis-light-induced superhydrophilicity of thin films

Miyauchi *et al.* reported that a WO_3_ thin film was formed by heating a precursor film at 500 °C for 30 min on a Pyrex glass substrate precoated with a SiO_2_ thin film of less than 100 nm thickness.^16^ The precursor film was formed by dropping a solution of dissolved H_2_WO_4_ into an aqueous ammonia solution. They observed a water contact angle reduction on the thin film to approximately 15° by irradiating it with Vis-light for more than 100 h from a normal fluorescent lamp containing UV-light of 0.01 mW cm^−2^ at 22 °C and a RH of 40%.

The reported angle was larger than that of the present F500 which was left under dark conditions for one day at almost the same relative humidity. Moreover, the corresponding angle on F500 was reduced significantly to a small angle of less than 10° by irradiating the light from a regular desk lamp, which mainly emits Vis-light and the same level of UV light as a fluorescent lamp, for only 72 h (three days) and in a RH range of 20–25%. Notably, the superhydrophilicity of the crystallized WO_3_ thin films appears only after 20 min of irradiation with Vis-light under identical conditions with the exception of RH (40–50%). Furthermore, the hydrophilicity of the thin films was not significantly reduced even if they were left in the dark for one day, and the superhydrophilicity could be easily restored by short-term Vis-light irradiation.

Takashima *et al.* reported that WO_3_ thin films with a thickness of 1 μm were formed by depositing amorphous WO_3_ films on silica glass substrates by reactive DC magnetron sputtering and annealing at temperatures between 300 and 800 °C for 1 h in air. They reported that the water contact angles of thin films annealed at 300–700 and 800 °C were reduced to less than 10° by irradiating with 1.0 mW cm^−2^ Vis-light for 30–120 and 20 min, respectively,^17^ although the ambient temperature and humidity were not stated. It was also reported that the large surface roughness (41.4 nm) of the thin film annealed at 800 °C contributed to the superhydrophilicity. On the other hand, F500, F600, and F700 had flat surfaces (Fig. 5) and very low haze values of 0.4–0.5%. Importantly, these films showed Vis-light-induced superhydrophilicity after only 20 min of irradiation, even though the light intensity used here was nearly 1/16 of that reported by Takashima *et al.* The present results suggest that it was the electronic state, which affects light excitation and the resulting interaction with water molecules, of the partially O-deficient WO_3_ thin films that was important rather than the surface roughness.

Wang *et al.* reported that the holes generated on the WO_3_ thin film surface by UV-light irradiation form oxygen vacancies that can adsorb hydroxyl groups on the film surface.^30^ Additionally, we previously reported that a rutile-type TiO_2_ thin film formed through a typical MPM process causes oxygen deficiency, which drastically improves the photocatalytic activity by Vis-light irradiation.^31^ Moreover, Wang *et al.* reported an increase in the photocatalytic activity of oxygen-deficient WO_3_ nanorods, which was obtained by adding citric acid to (NH_4_)_10_(H_2_W_12_O_42_)·*x*H_2_O.^32^ They demonstrated that surface oxygen vacancies, which act as photoelectron sinks, can capture photoelectrons and facilitate separation from their holes, resulting in high photocatalytic activity. Thus, surface oxygen vacancies of WO_3_ thin films are important for reactions under weak Vis-light.

Although the present WO_3_ thin films clearly exhibit Vis-light-induced superhydrophilicity, no content increase of the hydroxyl group on the surface was detected by XPS measurements in a vacuum. This is probably because the WO_3_ thin film originally has a large number of oxygen vacancies that can enhance hydrophilicity and weak Vis-light contributes to the adsorption of the surrounding H_2_O molecules.

It is clarified using the present WO_3_ thin films that water molecules in the atmosphere are incorporated into the photoinduced hydrophilization of WO_3_ thin films because the high RH drastically reduced the water contact angle. Photoelectrons captured by surface oxygen vacancies can easily react with several adsorbed water molecules at moderate RH levels in the range of 40–50%. Water molecules that react with electrons generate radicals and ions which facilitate network formation with the surrounding water molecules and increase the hydrophilicity of the thin film surface. The obtained results at a low RH of less than approximately 25% demonstrate that a network of water molecules can be formed; however, at a slower rate. Importantly, oxide ions are not embedded in the oxygen vacancies in this process, and even if the surface condition changes under dark conditions, superhydrophilicity can be quickly restored by irradiation with Vis-light.

## 5. Conclusions

A new tungsten complex salt in which a citrate ion coordinates to the central tungsten(vi) ion as a tridentate ligand was isolated, and the structure characterized through elemental analysis, IR, and NMR spectra was consistent with the results of thermal analysis of the powder. The ethanol solution in which the powder was dissolved was stable for a long time and could be spin-coated on a quartz glass substrate. The spin coated precursor film was heated in air at 500 °C or higher to obtain a pale blue transparent thin film with haze values of less than 0.5%. The thermal decomposition of the organic ligand and alkylamine in air effectively generated oxygen deficiencies in the WO_3_ thin film surfaces, resulting in pentavalent tungsten ions, as revealed by the XPS peak analysis.

The WO_3_ thin films with oxygen deficiencies have bandgap energies of 2.8–2.9 eV, film thicknesses of 100–120 nm, and smooth surfaces. A thermal analysis of the precursor complex indicated that the crystal phase transition occurs at around 600 °C. The strong adhesion of 49 MPa, of the thin film formed at 500 °C, on the glass substrate further increased when the temperature was raised.

The oxygen-deficient WO_3_ thin film exhibited superhydrophilicity by responding to a weak Vis-light of 0.06 mW cm^−2^. The duration of Vis-light irradiation for the thin film to become superhydrophilic is highly dependent on surrounding RH values. When the RH was in the range of 40–50%, superhydrophilicity appeared at a short-term irradiation time of 20 min. The hydrophilization level of the resulting superhydrophilic thin films decreased to some extent when they were left in the dark for one day. However, superhydrophilicity recovers easily by irradiation with Vis-light for a shorter time in comparison to when left in the dark. It was suggested by the comparison with WO_3_ films that the electronic state of the partially O-deficient WO_3_ thin film plays an important role in Vis-light-induced superhydrophilicity.

In this study, a tungsten oxide thin film was first formed using the MPM, in which a tungsten complex of citric acid was useful, and the resulting oxygen-deficient WO_3_ thin film responded sensitively to weak Vis-light. Therefore, the oxygen-deficient WO_3_ thin film formed using the MPM might also be applied to photocatalysts and oil separation^33,34^ working under Vis-light and with energy conversion materials.

## Author contributions

Taichi Murayama: conceptualization, formal analysis, investigation, writing – original draft, visualization. Mitsunobu Sato: conceptualization, methodology, writing – original draft, writing – review & editing, supervision, funding acquisition. Hiroki Nagai: conceptualization, methodology, investigation, writing – original draft, writing – review & editing, supervision, project administration, funding acquisition. Eiko Yasui: formal analysis, writing – review & editing.

## Conflicts of interest

There are no conflicts to declare.

## Supplementary Material

NA-005-D2NA00717G-s001
