# Supplementary material for: Visible-light-induced superhydrophilicity of crystallized WO3 thin films fabricated by using a newly isolated W6+ complex salt of citric acid
Source: Nanoscale Adv. 2023 Mar 7;5(7):1990–8. doi: 10.1039/d2na00717g (PMC10044916; doi:10.1039/d2na00717g)
Supplement: NA-005-D2NA00717G-s001 [file NA-005-D2NA00717G-s001.pdf]

Electronic Supplementary Material (ESI) for Nanoscale Advances.
This journal is © The Royal Society of Chemistry 2023

Visible-light-induced superhydrophilicity of crystallized WO_3 thin films fabricated by using a newly isolated W^{6+} complex salt of citric acid

Taichi Murayama[a], Mitsunobu Sato[b], Hiroki Nagai[a, b, *], and Eiko Yasui[c]

*nagai@cc.kogakuin.ac.jp

[a] Electrical and Electronics Engineering Program, Graduate School of Engineering, Kogakuin University of Technology and Engineering, Hachioji, Tokyo, 192-0015, Japan

[b] Department of Applied Physics, School of Advanced Engineering, Kogakuin University of Technology and Engineering, Hachioji, Tokyo, 192-0015, Japan

[c] Department of Chemistry and Life Science, School of Advanced Engineering, Kogakuin University of Technology and Engineering, Hachioji, Tokyo, 192-0015, Japan

SUPPORTING INFORMATION: S1 are the detail of Experimental. Fig. S1, Tables S1 and S2 support "3.1. Chemical characterization of dibutylammonium salt of W^{6+} complex with citrate ligands." Tables S3 and S4 support Fig. 3. Fig. S2 and S3 support "3. 3. Surface

morphology, film thickness, and adhesion strength of thin films”. Fig S2 and Table S5, S6 suppout Figure 4. Table S7 and S8 suppout 7 (a) and 7 (b), respectively.

S1. Experimental

S1. Materials

Citric acid (99.0%), dibutylamine (98.0%), and tungstic acid (99.9%) were purchased from FUJI-FILM Wako Pure Chemical Corporation (Miyazaki, Japan). Ethanol-d$_6$ (C_2D_5OD, 99 atom%) and deionized water were purchased from Tokyo Chemical Industry Co., Ltd. (Tokyo, Japan) and Kyoei Pharmaceutical Co., Ltd. (Chiba, Japan), respectively. Ethanol was purchased from Ueno Chemical Industries, Ltd. (Tokyo, Japan) and dried on 4A molecular sieves prior to use. All the other materials were used as received. Polished quartz glass (100 mm × 100 mm × 1.5 mm) was purchased from Akishima Glass Co., Ltd. (Tokyo, Japan). The quartz glass was cut to 20 mm × 20 mm × 1.5 mm and used as the substrate. The quartz glass substrates were sonicated with detergent in water for 30 min and rinsed twice with deionized water for 15 min in an ultrasonic bath to remove organic contaminants from the surfaces, followed by thorough rinsing with deionized water. Subsequently, the substrates were dried in an oven at 70°C.

S2. Isolation of dibutylammonium salt of W^{6+} complex with citrate ligands

11.64 g (60 mmol) of citric acid was disolved in 200 g of deionized water and stirred for 5 min, and 23.74 g (180 mmol) of dibutylamine was added to the solution and stirred for 5 min. 15.01 g (60 mmol) of tungstic acid was added to the reacted solution and the mixed solution was subsequently heated at 70°C for 2 h with continuous stirring. A small amount of the white powder was filtered using a paper filter, and the filtrate was evaporated to approximately 70 g under reduced pressure. The temperature of the solution with the newly precipitated white powder was maintained at 23°C overnight. The collected powder from the solution is denoted as PPRE.

S3. Preparation of WO_3 precursor solution

Into 3 g of ethanol, 0.78 g (0.94 mmol) of PPRE was dissolved. A clear precursor solution with the PPRE concentration of 0.25 mmol g^{-1} was prepared by stirring at 23°C for 1 h.

S4. Coating and heat treatment procedures

The precursor film was formed on a quartz glass substrate by dropping 100 μL of PS, spin-coating (1st step; 500 rpm for 5 sec, and 2nd step; 2000 rpm for 30 sec), and preheating at 70°C in a drying oven for 10 min. The precursor film was subsequently heat treated in an electric furnace 232FA (TOYO ROSHI KAISHA, Ltd., Tokyo, Japan) at 400, 500, 600 and

700°C for 30 min in air. The obtained thin films are denoted FT, where the T represents the hearing temperature.

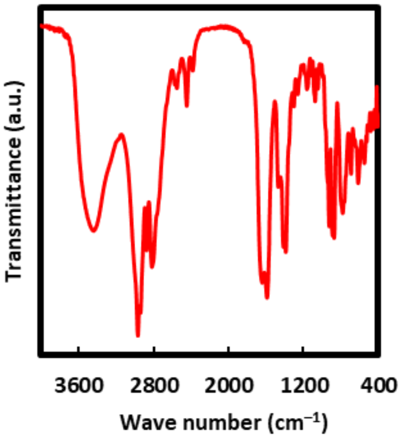

Fig. S1 FT-IR spectrum of PPRE. 200 mg of KBr as a diluent was ground in a mortar and compressed to form a pellet for reference. Separately, 3 mg of PPRE was mixed with 200 mg of pre-ground KBr. The mixed powders were thoroughly ground in a mortar and compressed to form sample pellets. Each pellet was measured in the range of 400–4000 cm^{-1} using 16 data acquisition methods.

Table S1 The chemical shift for the ^{1}H-NMR spectrum of PPRE in C_2D_5OD measured with tetramethylsilane as an internal standard (d = 0 ppm).

Chemical shift (ppm)	Signal multiplicity	Integral value	Assignment in ^{1}H-NMR spectrum
0	Singlet	-	Tetramethylsilane
0.96	Triplet	9H	CH_3 in $Bu_2NH_2^+$
1.11	Singlet	-	Ethanol-d_6
1.41	Sextet	6H	CH_2 linked to CH_3 in $Bu_2NH_2^+$
1.71	Quintet	6H	CH_2 linked only to CH_2 in $Bu_2NH_2^+$
2.62	Double doublet	2H	CH_2 in Hcit
2.96	Triplet	6H	CH_2 bonded to N in $Bu_2NH_2^+$
3.55	broad singlet	-	Ethanol-d_6
5.30	broad singlet	-	Ethanol-d_6

Table S2 The chemical shift for the ^{13}C-NMR spectrum of PPRE in C_2D_5OD measured with tetramethylsilane as an internal standard (d = 0 ppm).

Chemical shift (ppm)	Signal multiplicity	Assignment in ^{13}C-NMR spectrum
0	Singlet	Tetramethylsilane
13.99	Singlet	CH_3 in $Bu_2NH_2^+$
17.26	Septet	Ethanol-d_6
20.73	Singlet	CH_2 linked to CH_3 in $Bu_2NH_2^+$
28.84	Singlet	CH_2 linked to CH_2 in $Bu_2NH_2^+$
45.51	Singlet	CH_2 in Hcit
48.02	Singlet	CH_2 linked to N in $Bu_2NH_2^+$
56.80	Quintet	Ethanol-d_6
85.91	Singlet	C linked to OH in Hcit
177.37	Singlet	COO linked to CH_2 in Hcit
186.29	Singlet	COO linked to the tertiary C in Hcit

Dibutylammonium ion; $Bu_2NH_2^+$, Citrate ion; Hcit, Nitrogen atom; N, Carbon atom; C

Table S3 The peak position of XRD patterns in 20‑45° of the thin films obtained at several temperatures (F500, F600, and F700) and ICDD of triclinic (ICDD: No. 01–071–0305), monoclinic (ICDD: No. 01–083–0950) or orthorhombic (ICDD: No. 01–089–4477) WO_3.

	**2θ**							
**F500**	**23.46**	**24.37**		**28.96**	**34.10**	**35.64**	**41.77**	
**F600**	**23.50**	**24.46**	**26.85**	**29.07**	**34.27**	**35.77**	**41.90**	**44.42**
**F700**	**23.37**	**24.55**	**26.85**	**29.12**	**34.39**	**35.89**	**42.03**	**44.49**
**Triclinic**	**23.64**	**24.34**	**26.27**	**29.09**	**34.12**	**35.92**	**41.88**	**44.61**
**Monoclinic**	**23.58**	**24.37**	**26.59**	**28.93**	**34.17**	**35.65**	**41.89**	**44.70**
**Orthorhombic**	**23.47**	**24.25**	**26.47**	**29.40**	**34.01**	**35.73**	**41.73**	**44.56**

Table S4 The peak position of XRD patterns in 45–60° of the thin films obtained at several temperatures (F500, F600, and F700) and ICDD of triclinic (ICDD: No. 01–071–0305), monoclinic (ICDD: No. 01–083–0950) or orthorhombic (ICDD: No. 01–089–4477) WO_3.

	2θ							
**F500**	**45.59**	**47.35**		**49.68**	**50.50**	**53.85**	**55.59**	
**F600**	**45.81**	**47.54**		**49.93**	**50.77**	**54.00**	**55.82**	**58.16**
**F700**	**46.06**	**47.49**	**48.51**	**50.08**	**50.83**	**54.06**	**56.11**	**58.41**
**Triclinic**	**45.99**	**47.34**	**48.38**	**49.88**	**50.62**	**54.05**	**55.66**	**58.15**
**Monoclinic**	**45.74**	**47.25**	**48.25**	**49.93**	**50.72**	**54.15**	**55.62**	**58.17**
**Orthorhombic**	**45.46**	**46.92**	**48.25**	**49.93**	**50.72**	**54.15**	**55.66**	**58.17**

Table S5 The binding energies and the peak areas of deconvoluted W 4f and of O 1s peaks of F500, F600, and F700 before Vis-light irradiation.

Films	Binging energy (eV)						Peak area			O/W area ratio
	W^{5+}		W^{6+}		O^{2-}	OH		O 1s		
	$4f_{7/2}$	$4f_{5/2}$	$4f_{7/2}$	$4f_{5/2}$	O 1s		W^{6+} $4f_{7/2}$	O^{2-}	OH^{-}	
F500	34.8	35.7		37.4		531.8	25230	33060	4525	
F600	34.6	36.0	35.3		530.2	531.7	25796	33830	3064	2.90
F700	34.7	36.4		37.5		531.8	26204	34405	3074	

Table S6 The binding energies and the peak areas of deconvoluted W 4f and of O 1s peaks of F500, F600, and F700 after Vis-light irradiation at a RH range of 40–50% for 20 min.

Films	Binging energy (eV)						Peak area			O/W area ratio
	W^{5+}		W^{6+}		O^{2-}	OH		O^{2-}		
	$4f_{7/2}$	$4f_{5/2}$	$4f_{7/2}$	$4f_{5/2}$	O 1s		W^{6+} $4f_{7/2}$	O 1s	OH^{-}	
F500	34.7	35.7				531.8	26655	35019	1528	
F600	34.6	36.1	35.3	37.4	530.3	531.7	25691	33715	1382	2.90
F700	34.7	36.3					25431	33380	2251	

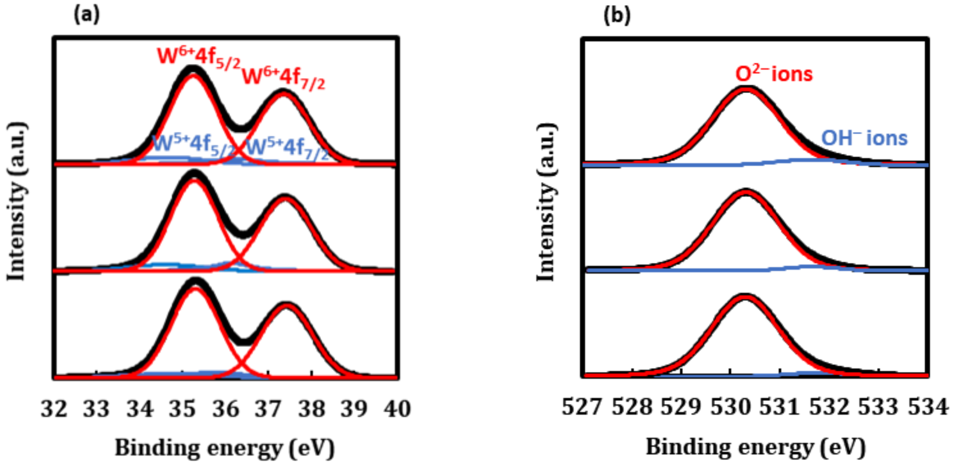

Fig. S2 High-resolution XPS spectra of (a) W 4f, (b) O 1s of the thin films obtained by heating at several temperatures (F500 (Bottom lines), F600 (Middle lines), and F700 (Top lines)) after Vis-light irradiation at a RH range of 40–50% for 20 min. The thick bold line indicates the original XPS data, whereas the colored line indicates the theoretical fitted curve assuming a Voigt distribution.

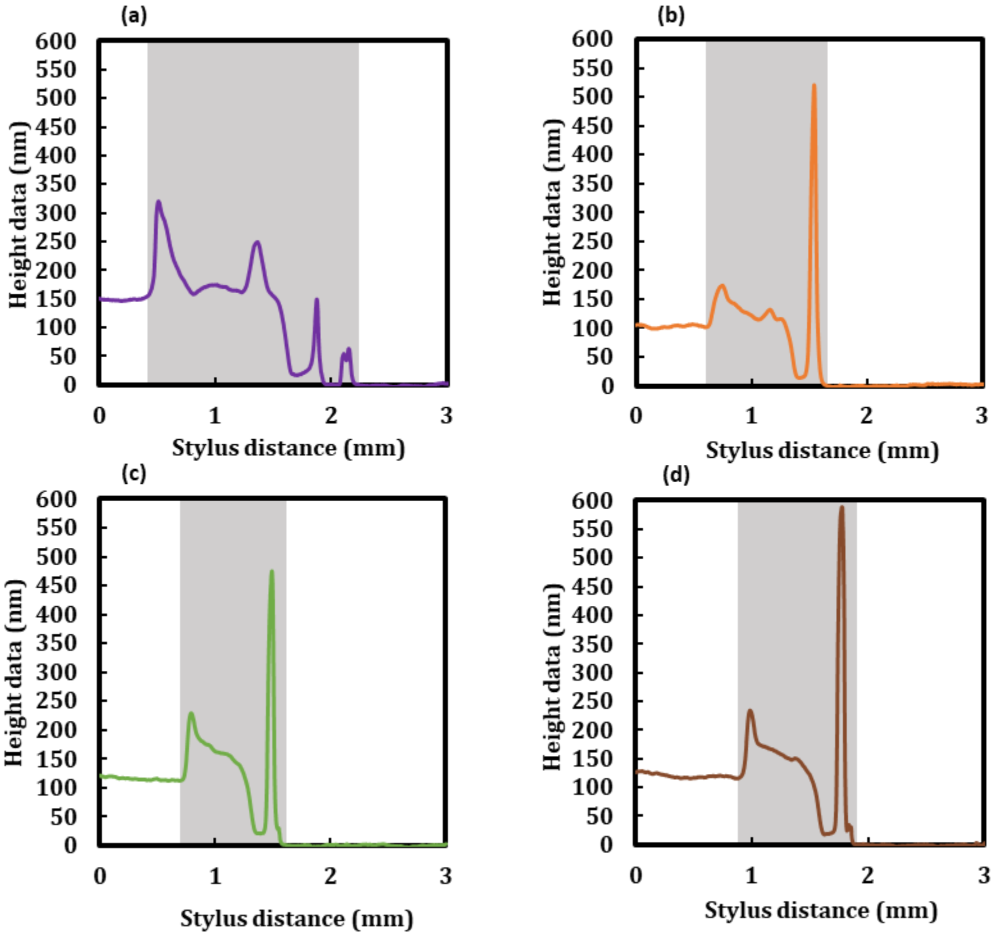

Fig. S3 The hysteresis curve of film thickness for the thin films obtained by heating at several temperatures (a) F400, (b) F500, (c) F600, and (d) F700. The film thickness is the difference between the height of two white backgrounds (light; film, right; substrate).

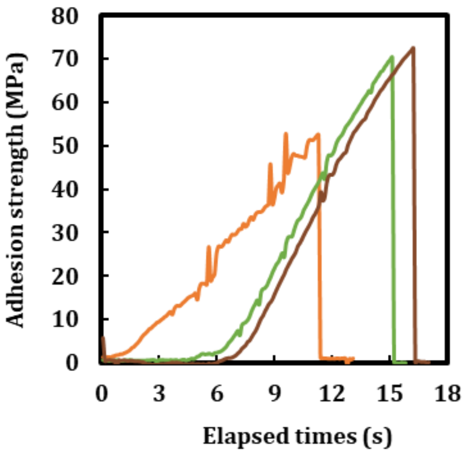

Fig. S4 The hysteresis curve of adhesion strengths for F500, F600, and F700 on the quartz glass substrate was shown. The adhesion strength was examined using a stud-pull adhesion test. A stand pin, P/N901106, with an internal diameter of 2.7 mm was attached to the film using epoxy glue and placed in an oven at 150°C for 1 h. The test was subsequently performed by pulling the stand pin with a load ranging from 0 to 100 kg at [illegible]

Table S7 Photographs of water contact angles for the thin films obtained by heating at several temperatures F400, F500, F600, and F700 after 1, 2, and 3 days of irradiation to visible light at temperatures ranging from 20–25°C and humidity ranging from 20–25%, and after 1 day of storage in the dark at the same temperature and humidity.

Films	Before Vis-light irradiation	After visible-light irradiation			After 1-day storage in the dark
		1 day	2day	3 day	
F400	62° ± 1° 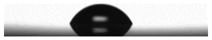	55° ± 1° 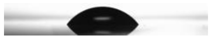	55° ± 1° 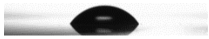	56° ± 1° 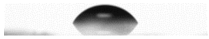	55° ± 1° 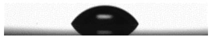
F500	61° ± 4° 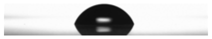	17° ± 1° 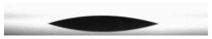	10° ± 1° 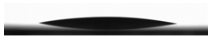	7° ± 1° 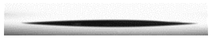	31° ± 2° 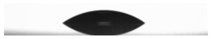
F600	64° ± 4° 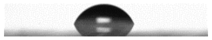	18° ± 9° 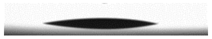	9° ± 4° 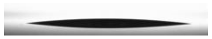	7° ± 1° 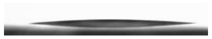	32° ± 2° 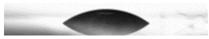
F700	61° ± 1° 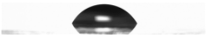	21° ± 11° 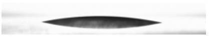	13° ± 10° 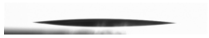	10° ± 7° 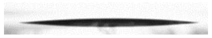	33° ± 1° 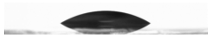

Table S8 Photographs of water contact angles for the thin films obtained by heating at several temperatures F400, F500, F600, and F700 after 10, 20, and 30 minutes of irradiation to visible light at temperatures ranging from 20–25°C and humidity ranging from 40–50%, and after 1 day of storage in the dark at the same temperature and humidity.

Films	Before Vis-light irradiation	After visible-light irradiation				After 1-day storage in the dark
		10 min	20 min	30 min	60 min	
F400	70° ± 3° 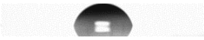	57° ± 4° 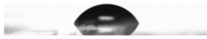	54° ± 2° 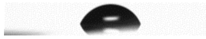	51° ± 2° 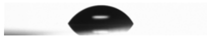	49° ± 3° 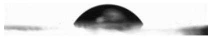	56° ± 3° 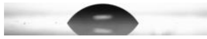
F500	23° ± 3° 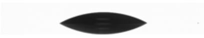	11° ± 2° 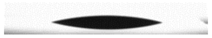	8° ± 1° 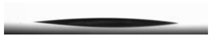	7° ± 1° 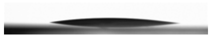	5° ± 1° 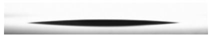	12° ± 1° 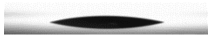
F600	23° ± 2° 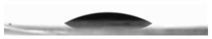	8° ± 1° 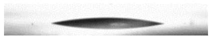	6° ± 1° 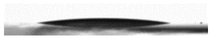	5° ± 1° 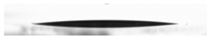	5 ± 1° 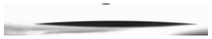	13° ± 1° 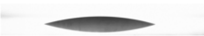
F700	24° ± 1° 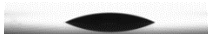	8° ± 1° 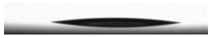	6° ± 1° 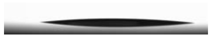	5° ± 1° 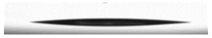	4° ± 1° 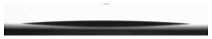	13° ± 1° 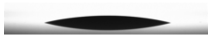
